# Explainer: Stochastic extinction

**DOI:** 10.1017/ext.2026.10023

**Published:** 2026-10-15

**Authors:** Raymond L. Tremblay

**Affiliations:** Biology, University of Puerto Rico, Puerto Rico

**Keywords:** demographic stochasticity, functional extinction, mass extinction, species extinction

## Abstract

*Stochastic extinction* is extinction caused by chance fluctuations (in births and deaths, in gene frequencies, and in the environment, including its rare extremes) rather than by a deterministic decline to zero. The phrase is an umbrella over two classes of randomness: environmental fluctuation, which is external to the population and whose rare extremes (“catastrophes”) are the tail of that same distribution rather than a separate class; and demographic accidents, which are internal and unavoidable in finite numbers, comprising the vagaries of birth and death, and the loss of genetic variability. It also covers a separate palaeontological usage in which “stochastic” instead means extinction *non-selective with respect to traits.* Most productively it is a population-level idea: even a population with, on average, a positive or stable growth rate can be lost to chance once it becomes small, so that stochastic extinction is best read as a probability: the chance, over a stated horizon, that a self-replacing population completing its full life cycle in the wild is no longer attainable. Whether a given decline is labelled “stochastic” or “deterministic” frequently depends on the scale and time horizon of observation rather than on the biology itself. The term therefore requires four things to be stated: the source of randomness, the biological scale (individual, population, metapopulation or clade), the time horizon and whether the claim concerns an underlying *process* or an observed *pattern.*

## Graphical abstract

**Figure:**
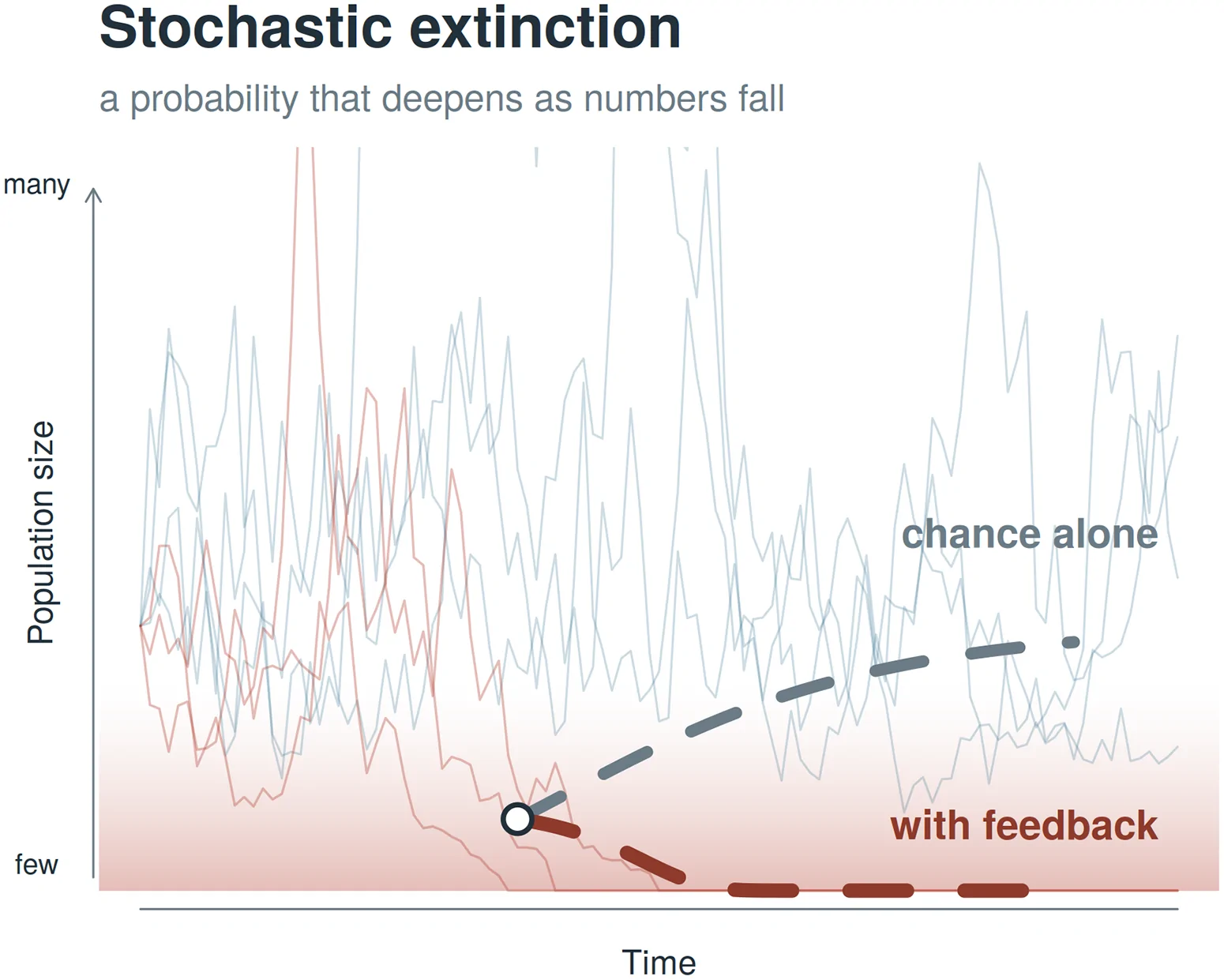

## Impact statement

Conservation decisions are often taken as though a species’ fate were settled by its average trend: stable numbers mean safety, falling numbers mean something is driving them down. Both readings can mislead. A small population can be lost while its average tendency is stable, and a population that appears to be declining may be in the trough of a fluctuation that will reverse. Whether an extinction is called "stochastic" therefore carries practical weight, because it decides whether managers look for a cause to remove or for numbers to rebuild.The difficulty is that the phrase is used for several different things: noise in births and deaths, variation in the environment, the erosion of genetic variability, and, in palaeontology, extinction that simply does not sort species by their traits. This Explainer separates those meanings and sets out the four things a careful use of the term must specify. It is written for readers who meet the phrase from outside extinction research (journalists, policymakers, students and practitioners) and who need to know what is, and is not, being claimed.

## The idea

Most extinctions are frequently pictured as deterministic: a population undergoes a sustained, directional decline (habitat loss, overharvest, a changing climate) until it reaches zero. Stochastic extinction is the complement. It is the recognition that even a population with, on average, a positive or stable growth rate can disappear through the accumulation of chance events. Births and deaths, good and bad years, the shuffling and loss of genes and rare severe years are all probabilistic. A population that is only holding steady or slowly declining is, over a long enough interval, certain to be extinguished this way; even one growing on average is not immune: it retains a non-zero probability of being driven to zero by a run of bad luck (Feller, 1939). Feller’s result, which already contains the whole idea, is stated in full in Supplementary Section S1.

**Figure 1.**
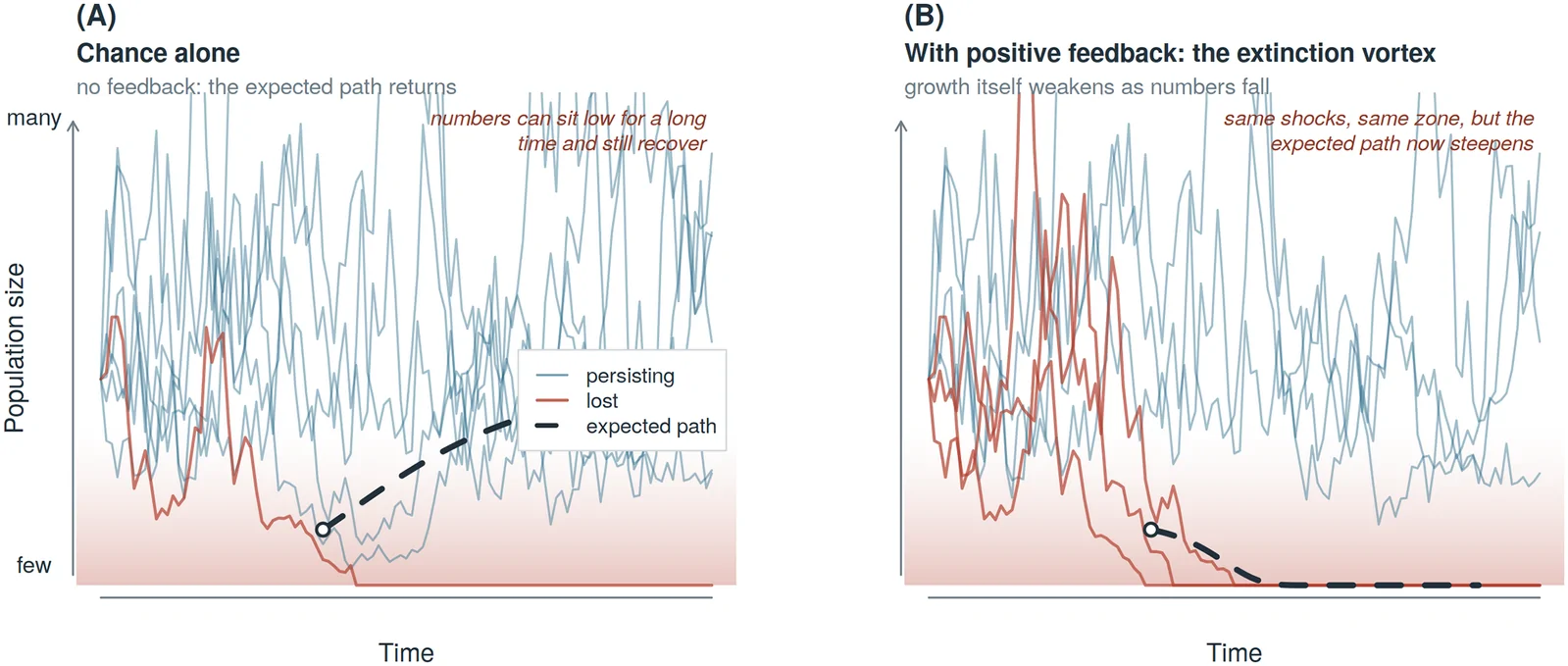
Stochastic extinction as a chance-driven loss of viability. Simulated trajectories of a small population with, on average, a stable growth rate. Both panels use the same random shocks, so the difference between them is the biology, not the noise. (A) With chance alone the trajectories wander; numbers can sit low for a long time and still recover, and the expected path of a population that has entered the shaded region (heavy dashed line) returns. (B) When growth itself weakens as numbers fall, because mate-finding failure, sex-ratio failure and lost genetic variability reinforce one another, that expected path steepens instead: the extinction vortex. Neither panel contains a line that gets crossed. The shading marks a risk that deepens as numbers fall, at a rate neither constant nor predictable across densities. The vertical axis is qualitative (few to many). Figure 1. long description.

It is tempting to picture stochastic extinction as the last individual dying at random, the final shot in a *field of bullets.* That image captures a real but different sense of the word, used mainly in palaeontology (see below). In population biology the important point is earlier and more practical: chance dominates the fate of a lineage long before the last individual, once the population becomes small enough that random variation outweighs its average tendency to grow. The expected time to extinction lengthens steeply with population size, so small populations are far more vulnerable than large ones (MacArthur and Wilson, 1967) (Figure 1).

## An umbrella, not one concept

Conservation biology inherited a partition of the sources of randomness, named explicitly by Shaffer (1981) and formalised by Lande (1993). Two of its divisions are better collapsed, leaving what is external to the population and what is internal to it:

- Environmental fluctuation: year-to-year variation in vital rates driven by a fluctuating environment, which can threaten even large populations (Lande, 1993). Rare, severe events belong here too. Setting “catastrophes” apart as a class of their own begs the question of what counts as one, and there is no scale-free answer: a population’s measured variability keeps rising with the length of the census, so extremes appear at every scale of observation (Inchausti and Halley, 2001; Pimm and Redfearn, 1988), which is also why recovery from a disturbance is hard to delimit and hard to measure (Pimm et al., 2019). A catastrophe is the tail of the environmental distribution, not a different kind of thing.
- Demographic accidents: the unavoidable consequences of finite numbers, in three forms: the *vagaries of death* (who dies, when individuals die independently), the *vagaries of birth* (how many young appear, and of which sex) and the *loss of genetic variability* through genetic drift and inbreeding, at a rate set by the effective population size. They matter most in small populations (Melbourne and Hastings, 2008). The first two can be modelled exactly; the third cannot, because how a given loss of variation translates into survival and reproduction is still poorly known and has to be established case by case (Pimm et al., 2006). Analyses that model only the first two therefore understate risk (Kong et al., 2021). The three are not independent, which is why they belong together: as numbers fall each strengthens the others, so the growth rate itself declines, producing the *extinction vortex* (Fagan and Holmes, 2006; Gilpin and Soulé, 1986).

**Figure 2.**
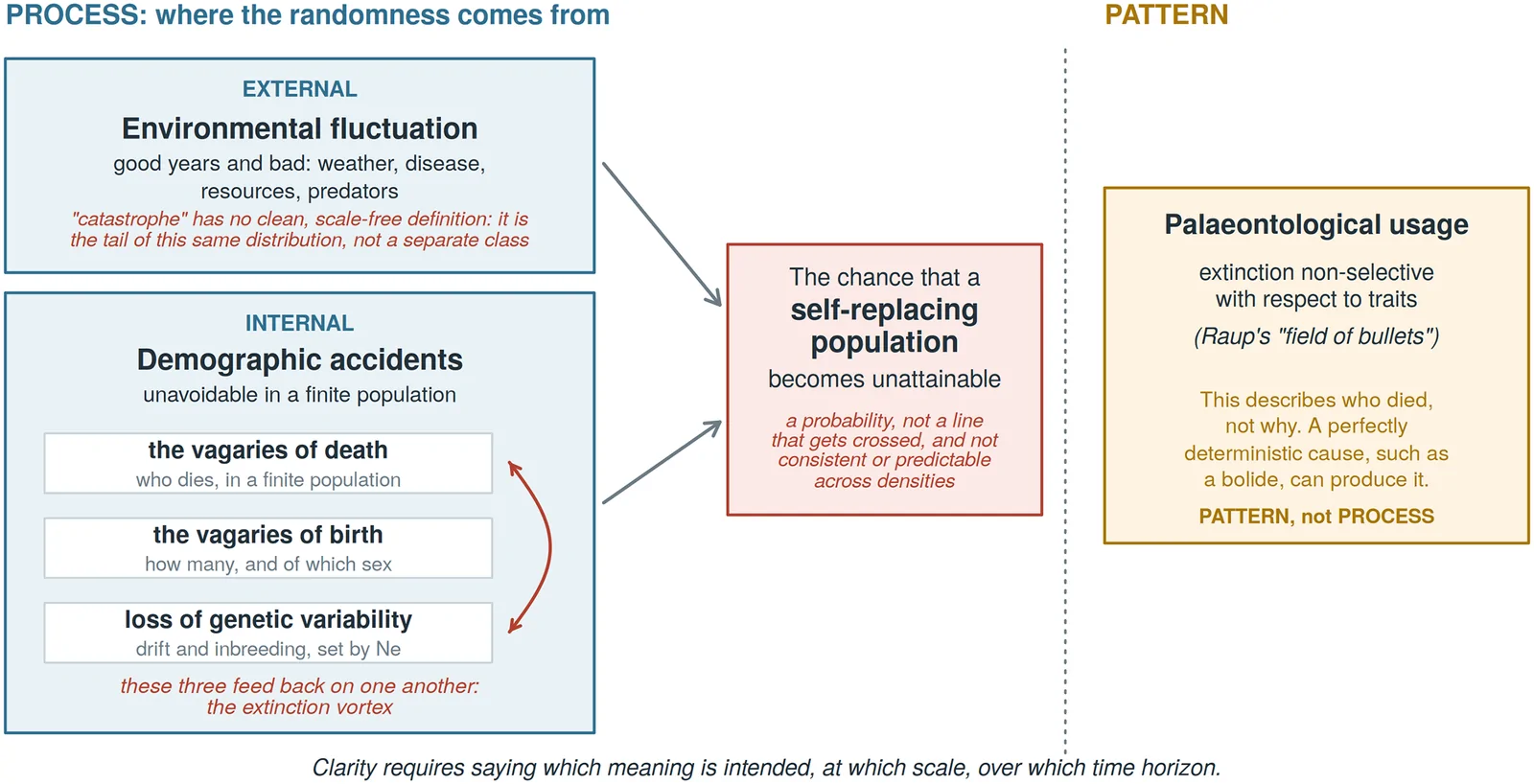
One term, several meanings. The two population-level classes of randomness (environmental fluctuation, external to the population, and demographic accidents, internal to it) act on the process of population change and together set the chance that a self-replacing population is no longer attainable. The palaeontological usage, extinction non-selective with respect to traits (Raup’s “field of bullets”), describes an observed pattern and can be produced even by a deterministic cause. Clarity requires saying which meaning, at which scale, over which horizon, is intended. Figure 2. long description.

A third, conceptually different usage comes from palaeontology. There “stochastic” extinction usually means extinction that is non-selective with respect to traits, Raup’s *field of bullets*, in which who dies is unrelated to morphology, ecology or body size (Raup, 1991). Crucially, this describes a *pattern* and is compatible with a perfectly *deterministic* cause: a bolide impact is not random, yet it can kill clades at random with respect to their traits. The two classes above, by contrast, describe a *process.* Conflating the two, pattern versus process, is a principal source of confusion in how the term is used (Figure 2).

## A population-biology reading: When viable populations are not attainable

**Figure 3.**
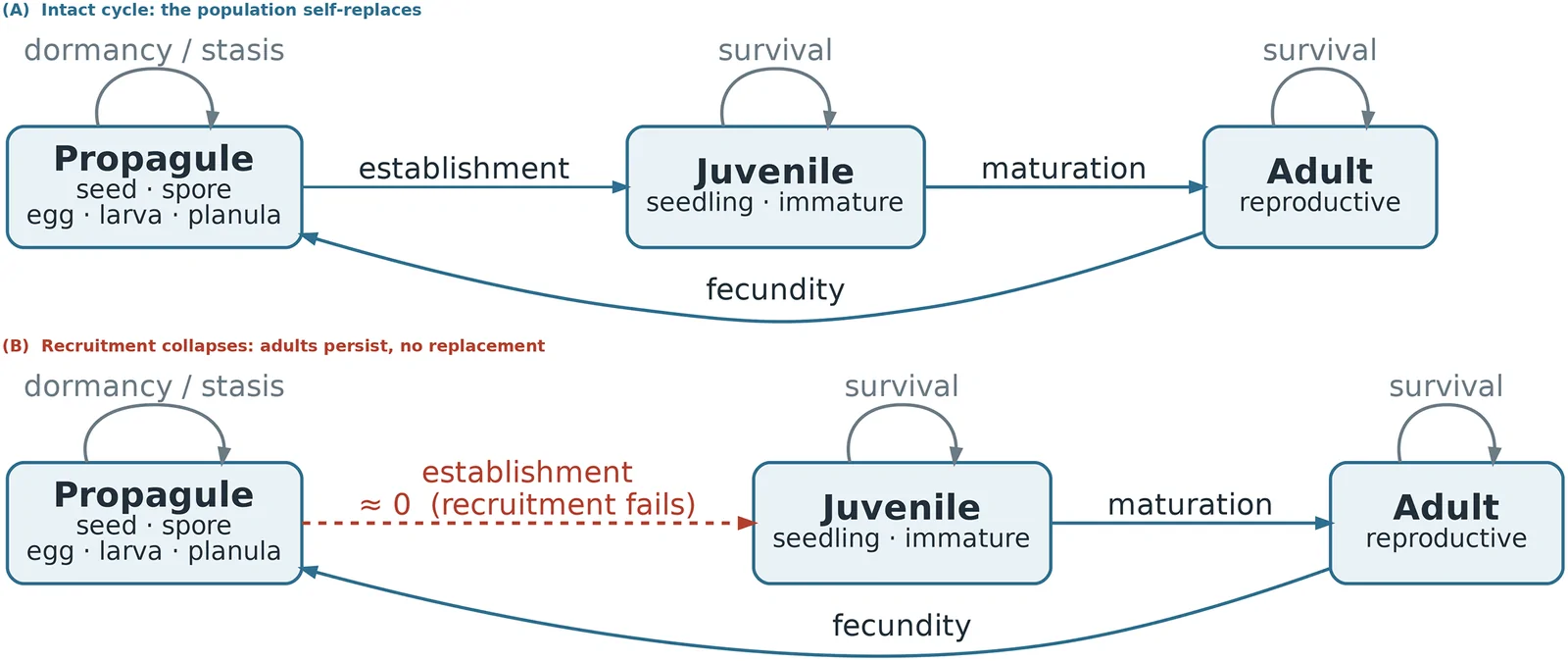
Stochastic extinction as failure to complete the life cycle. A taxon-neutral, stage-structured life cycle (read each stage for your organism: seed, spore, egg, larva, or planula). (A) When every transition needed to close the cycle is intact, the population self-replaces. (B) When recruitment collapses (establishment falls to near zero because mates, pollinators, or symbiotic partners are lost), adults persist and still produce propagules, but the cycle cannot close and the population can no longer self-replace. Crucially, the presence of all life-cycle stages does not guarantee viability: a population can contain every stage, adults included, yet still be unable to replace itself. Stochastic extinction, in this view, is the chance-driven weakening of any transition needed to close the cycle, and the limiting process can act at any stage or transition. Establishment is only the example shown here; survival, maturation, or fecundity can equally be the failing step. Read each stage for your organism: a propagule is a seed (plants), spore (fungi), egg or larva (animals and fish), or planula (corals); propagule dormancy/stasis applies chiefly to seed and spore banks, and many propagules (eggs, larvae, planulae) lack a persistent stage. Figure 3. long description.

For a working population biologist, the most useful reading is operational and scale-explicit. A lineage is effectively lost not at the death of its last individual but when the probability, over a stated horizon, that a self-replacing population completing its full life cycle in the wild remains attainable has become small (Figure 3). Standing adults that cannot recruit, because mates, pollinators, or symbiotic partners have become too rare, are already on a one-way trajectory, even when census numbers look reassuring. This is the regime in which stochastic processes become decisive: Allee effects, of which mate-finding failure is one mechanism, amplify demographic accidents. Viewed this way, stochastic extinction is a chance-driven loss of viability rather than the crossing of a line, and it sits naturally alongside the concept of *functional extinction* (Jarić, 2015).

This reading is deliberately conceptual rather than tied to any single regulatory definition, and it is not without tension. Reserving the word “extinct” for the disappearance of the last individual is more conservative and more easily audited; treating a lineage as effectively extinct once viability is unattainable is more biologically honest about commitment to loss but harder to apply at the moment of classification. The disagreement is real and worth stating plainly: it is, at root, a disagreement about *scale and time horizon*, not about whether chance matters.

## Why precision matters

Because the boundary between “stochastic” and “deterministic” shifts with scale and time horizon, it is often a methodological choice rather than a property of nature. A drought is an environmental driver (deterministic) to a demographer modelling one population and a random shock (stochastic) to a metapopulation model that does not resolve weather. Neither is wrong; they describe different levels of the same system.

Clear use of *stochastic extinction* therefore means specifying, every time: (1) which source of randomness is meant; (2) the biological scale (individual, population, metapopulation, or clade); (3) the time horizon over which the claim holds and (4) whether the statement is about an underlying process or an observed pattern. With those four coordinates fixed, the term is precise and powerful. Without them, it is one of the most ambiguous phrases in extinction research.

*The historical development of the concept, from Feller’s birth-and-death processes to MacArthur and Wilson, Shaffer, Lande, and the palaeontological tradition, together with the supporting literature and a fuller treatment of scale, is given in the Supplementary Material.*

## Supporting information

10.1017/ext.2026.10023.sm001 Tremblay supplementary material Tremblay supplementary material

## Data Availability

No data were generated or analysed in this article. Figure 1 illustrates a simulated example and has no underlying dataset; Figures 2 and 3 are schematic.

## Supplementary material

The supplementary material for this article can be found at http://doi.org/10.1017/ext.2026.10023.

## Long descriptions

### Figure 1 Long description

The figure has two panels side by side, labelled A and B. Both share the same axes. The vertical axis is labelled Population size and is qualitative, running from few at the bottom to many at the top. The horizontal axis is labelled Time and has no units. Both panels are driven by the same sequence of random shocks, so the only difference between them is the biology.

In each panel a band of red shading rises from the baseline and fades out towards the middle of the panel. The shading deepens continuously towards zero and has no boundary line at any height.

A legend in panel A identifies three line types. Pale blue grey lines are populations that persist. Red lines are populations that are lost. A heavy black dashed line is the expected path of a population that has entered the shaded region, and a small open circle marks where that expected path begins.

Panel A is headed Chance alone, with the subheading no feedback: the expected path returns. Most trajectories are pale blue and fluctuate irregularly across the upper part of the panel from start to finish. One red trajectory descends through the shaded region, reaches zero part way along the time axis, and then runs flat along the baseline. The heavy dashed expected path starts low inside the shaded region and rises towards the right. An annotation in the upper right reads: numbers can sit low for a long time and still recover.

Panel B is headed With positive feedback: the extinction vortex, with the subheading growth itself weakens as numbers fall. The same shocks give a different outcome. Three red trajectories descend into the shaded region and reach zero, at three different times. The heavy dashed expected path starts at the same open circle position as in panel A, but falls to the baseline and stays there. An annotation in the upper right reads: same shocks, same zone, but the expected path now steepens.

The visual comparison is between the two heavy dashed paths. From the same starting position, that path rises in panel A and falls to zero in panel B. Neither panel contains a threshold line.

Navigate back to Figure 1.

### Figure 2 Long description

The figure is a diagram divided by a vertical dashed line into a wide left portion and a narrower right portion. The left portion is headed PROCESS: where the randomness comes from. The right portion is headed PATTERN.

The left portion contains two blue outlined boxes, one above the other.

The upper box is labelled EXTERNAL and then, in bold, Environmental fluctuation, with the gloss good years and bad: weather, disease, resources, predators. A note inside the box reads: catastrophe has no clean, scale free definition, it is the tail of this same distribution, not a separate class.

The lower box is labelled INTERNAL and then, in bold, Demographic accidents, with the gloss unavoidable in a finite population. Inside it are three white sub boxes, one above the other. The first reads the vagaries of death, glossed who dies, in a finite population. The second reads the vagaries of birth, glossed how many, and of which sex. The third reads loss of genetic variability, glossed drift and inbreeding, set by Ne. A curved double headed arrow runs down the right hand side of the three sub boxes, and a note beneath them reads: these three feed back on one another, the extinction vortex.

One arrow leaves each of the two blue boxes. Both arrows converge on a red outlined box at the centre of the figure, which reads: The chance that a self replacing population becomes unattainable. A note inside that box reads: a probability, not a line that gets crossed, and not consistent or predictable across densities.

To the right of the dashed line stands a single gold outlined box headed Palaeontological usage. It reads: extinction non selective with respect to traits, Raup’s field of bullets. It continues: This describes who died, not why. A perfectly deterministic cause, such as a bolide, can produce it. In bold at the foot of that box: PATTERN, not PROCESS. No arrow joins this box to anything on the left of the dashed line.

A single line of text runs across the foot of the whole figure: Clarity requires saying which meaning is intended, at which scale, over which time horizon.

Navigate back to Figure 2.

### Figure 3 Long description

The figure has two panels, one above the other. Each panel shows the same life cycle diagram, built from three rounded pale blue boxes arranged left to right and joined by arrows.

The left box reads Propagule, with the gloss seed, spore, egg, larva, planula. The middle box reads Juvenile, with the gloss seedling, immature. The right box reads Adult, with the gloss reproductive.

Above each box is a curved arrow that loops back to the same box. The loop above Propagule is labelled dormancy or stasis. The loops above Juvenile and above Adult are both labelled survival.

A straight arrow runs from Propagule to Juvenile, labelled establishment. A second straight arrow runs from Juvenile to Adult, labelled maturation. A long curved arrow runs back from Adult to Propagule beneath the three boxes, labelled fecundity. Together these close the cycle.

The upper panel is labelled A and headed Intact cycle: the population self replaces. Every arrow in it is solid.

The lower panel is labelled B and headed, in red, Recruitment collapses: adults persist, no replacement. It is identical to panel A with one change. The establishment arrow from Propagule to Juvenile is now red and dashed, and its label, also in red, reads establishment approximately zero, recruitment fails. Every other arrow stays solid, including adult survival and fecundity, so adults are still present and still producing propagules while the loop cannot close.

Navigate back to Figure 3.

## References

[r1] Fagan WF and Holmes EE (2006) Quantifying the extinction vortex. Ecology Letters 9(1), 51–60. 10.1111/j.1461-0248.2005.00845.x. 16958868

[r2] Feller W (1939) Die Grundlagen der Volterraschen Theorie des Kampfes ums Dasein in wahrscheinlichkeitstheoretischer Behandlung. Acta Biotheoretica 5(1), 11–40. 10.1007/BF01602932.

[r3] Gilpin ME and Soulé ME (1986) Minimum Viable Populations: Processes of Species Extinction. In Conservation Biology: The Science of Scarcity and Diversity. Sunderland, Massachusetts: Sinauer Associates.

[r4] Inchausti P and Halley J (2001) Investigating long-term ecological variability using the global population dynamics database. Science 293(5530), 655–657. 10.1126/science.293.5530.655. 11474102

[r5] Jarić I (2015) Complexity and insidiousness of cryptic function loss mechanisms. Trends in Ecology & Evolution 30(7), 371–372. 10.1016/j.tree.2015.04.014. 26001623

[r6] Kong L, Xu W, Xiao Y, Pimm SL, Shi H and Ouyang Z (2021) Spatial models of giant pandas under current and future conditions reveal extinction risks. Nature Ecology & Evolution 5(9), 1309–1316. 10.1038/s41559-021-01520-1. 34312523

[r7] Lande R (1993) Risks of population extinction from demographic and environmental stochasticity and random catastrophes. The American Naturalist 142(6), 911–927. 10.1086/285580. 29519140

[r8] MacArthur RH and Wilson EO (1967) The Theory of Island Biogeography. Princeton, NJ: Princeton University Press.

[r9] Melbourne BA and Hastings A (2008) Extinction risk depends strongly on factors contributing to stochasticity. Nature 454(7200), 100–103. 10.1038/nature06922. 18596809

[r10] Pimm SL, Dollar L and Bass OL (2006) The genetic rescue of the Florida panther. Animal Conservation 9(2), 115–122. 10.1111/j.1469-1795.2005.00010.x.

[r11] Pimm SL, Donohue I, Montoya JM and Loreau M (2019) Measuring resilience is essential to understand it. Nature Sustainability 2(10), 895–897. 10.1038/s41893-019-0399-7. PMC6923136 31858022

[r12] Pimm SL and Redfearn A (1988) The variability of population densities. Nature 334(6183), 613–614. 10.1038/334613a0.

[r13] Raup DM (1991) Extinction: Bad Genes or Bad luck? New York: W. W. Norton. 11540932

[r14] Shaffer ML (1981) Minimum population sizes for species conservation. Bioscience 31(2), 131–134. 10.2307/1308256.

