# Supplementary material for: Explainer: Stochastic extinction
Source: Camb Prism Extinct. 2026 Oct 15;4:e16. doi: 10.1017/ext.2026.10023 (PMC13748990; doi:10.1017/ext.2026.10023)
Supplement: Supplementary file 1 [file S2755095826100230sup001.pdf]

Supplementary Material for Explainer: Stochastic extinction

The story behind the idea, and the supporting literature

2026-08-25

Explainer: Stochastic extinction

This supplement expands on the two-page Explainer in three ways: it traces the **genealogy of the concept** through the history of biology; it develops the parts that the main text could only state, in particular the **scale (unit-of-reference) problem**; and it provides an **annotated guide to the key literature**.

S1. A short history of an old idea

The notion that extinction is fundamentally a matter of chance is older than conservation biology and older than the phrase "stochastic extinction" itself. It arrived in biology from probability theory and was applied to disease and to abstract populations before it was ever applied to endangered species (Figure S1). Table S1 sets out the same sequence in full, including the contributions the figure has no room to mark.

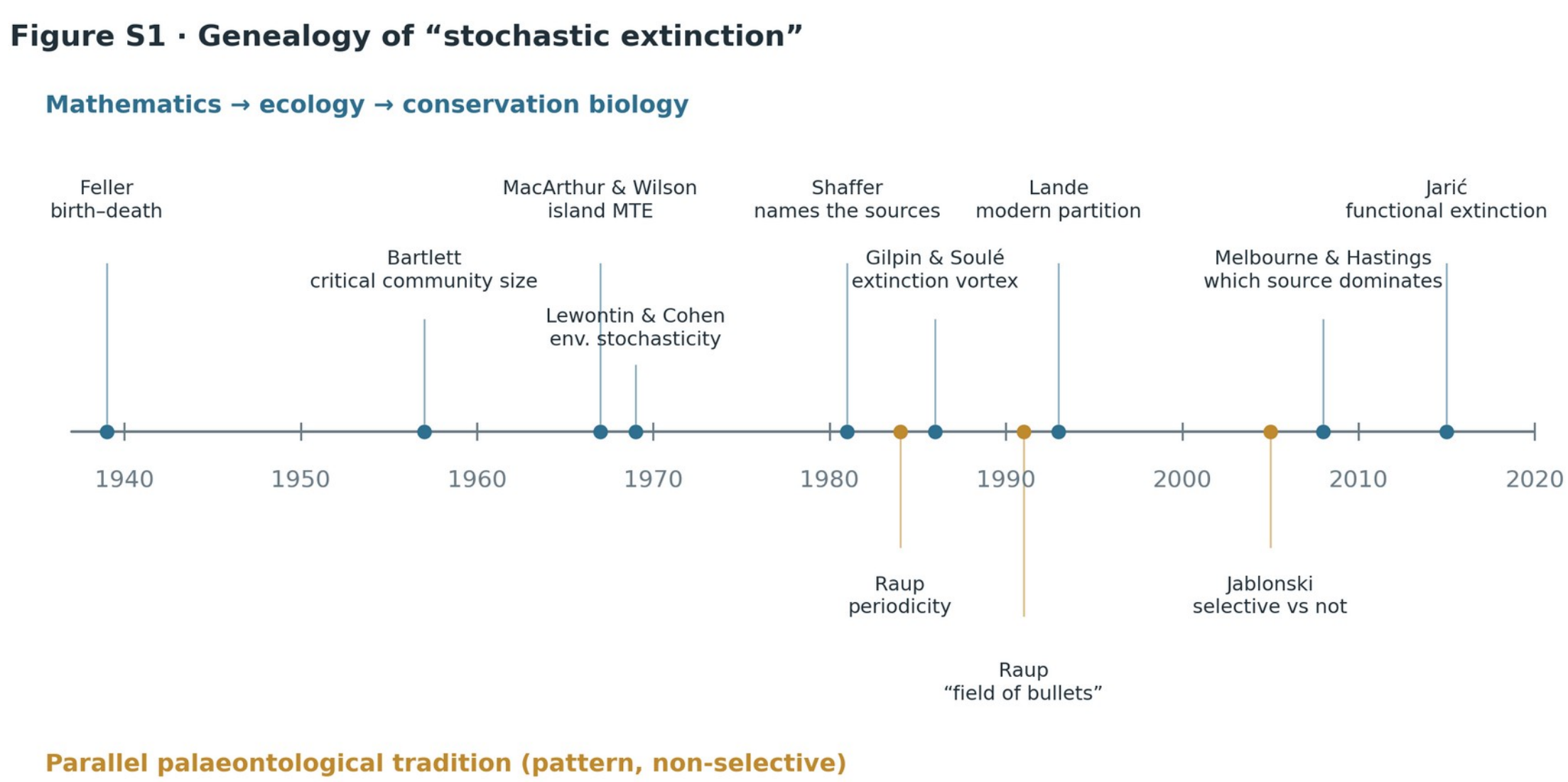

Figure S1. Genealogy of "stochastic extinction." *A timeline of the concept from its mathematical origins (birth-and-death processes, critical community size) through its entry into ecology (MacArthur & Wilson; Lewontin & Cohen) and its naming and formalisation in conservation biology (Shaffer; Gilpin & Soulé; Lande), shown alongside the parallel palaeontological tradition, in which "stochastic" carries two distinct senses, a random-timing (periodicity) sense (Raup 1984) and extinction non-selective with respect to traits, the field of*

bullets (Raup 1991). The two traditions developed largely independently, which is a principal reason the term is ambiguous today.

Mathematical origins (1930s–1950s). The conceptual ancestor of the entire literature is Feller (1939), who recast Lotka–Volterra population dynamics as a *birth-and-death process*, a stochastic model in which the probability of ultimate extinction emerges as a natural property of the system.

Feller's result deserves to be stated precisely, because it already contains the whole idea of stochastic extinction (his paper is in German). Modelling population size as a continuous stochastic process, he lets denote the mean per-capita growth tendency, a measure of the statistical spread of the fluctuations about it, and the initial size. He then derives the probability that the population is extinct by time . Crucially, unlike the deterministic or strictly linear picture (in which a growing population can never reach zero), this probability is **always positive**. Taking the limit gives the probability that the population *ever* goes extinct (Feller, 1939):

In words: a population whose mean growth tendency is zero or negative () is *certain* to go extinct eventually; but even a population with a strictly **positive** mean growth rate () carries a non-zero probability of ultimate extinction, , small when the population is large or its fluctuations are mild, yet never zero. Extinction is possible at all only because of the variance : with no fluctuations, a population with positive mean growth could never die out. This is the mathematical root of the Explainer's central claim that chance alone can extinguish a lineage that is, on average, holding its own, or even growing.

Kendall (1948; see also Kendall, 1949) gave the complete solution of the generalised birth-and-death process, one with time-dependent rates, and, for that general case, the first explicit expression for the probability of ultimate extinction. Bartlett (1949; and Bartlett, 1960) unified these ideas across ecology and epidemiology.

A point worth emphasising for the Explainer's central argument: the *life-cycle / viability* reading of stochastic extinction is not a modern, PVA-era invention. It is present at the very origin. Bartlett (1957) introduced the **critical community size**, the population threshold below which measles cannot maintain its transmission cycle and stochastically *fades out*. That is, in all but name, a viability threshold for a pathogen's life cycle, described three decades before Shaffer (1981).

Entry into ecology (1960s–1970s). MacArthur and Wilson (1967) gave stochastic extinction its first major ecological application, treating the extinction of an island population as a stochastic process and deriving a mean time to extinction that scales steeply with population size. Lewontin and Cohen (1969) established the foundation for *environmental* stochasticity specifically, showing that it is the geometric (not arithmetic) mean growth rate that governs long-term fate when growth rates vary randomly, so that a population can have a positive mean growth rate and still face certain extinction. Richter-Dyn and Goel (1972; and the textbook synthesis of Goel and Richter-Dyn, 1974) bridged abstract birth-death theory and applied population biology.

Naming the sources (1980s). Shaffer (1981) explicitly named and partitioned the sources of stochasticity (demographic, environmental, genetic, and catastrophic) that structure the field to this day, in the service of defining minimum viable populations. Gilpin and Soulé (1986) coined the **extinction vortex**, the feedback by which small size, stochasticity, and genetic deterioration reinforce one another.

Mean lifetimes and the amplitude of fluctuation (1981). Alongside this naming of sources, the theory of population *lifetimes* advanced. Leigh (1981) derived an approximate formula for the average time to extinction, the mean lifetime, of a population in a continuously varying environment, in terms of its initial size, its equilibrium size, its intrinsic capacity for increase, and the impact of environmental variation. His central qualitative result anticipates the modern partition: when the only randomness is the demographic accident of finite size (a *constant* environment, in the MacArthur & Wilson sense), mean lifetime rises steeply with size; but under environmental variation it increases far more slowly, and is governed chiefly by the **relative amplitude** of a population's fluctuations rather than by how large the population is. Abundance alone therefore cannot buy long persistence in a fluctuating world, the qualitative demographic-versus- environmental contrast that Lande (1993) would render exact a decade later.

Quantitative formalisation (1990s–2010s). Lande (1993) gave the canonical treatment of extinction risk from demographic and environmental stochasticity plus random catastrophes, and is the standard citation for the modern partition. Grimm and Wissel (2004) unified persistence measures through the *intrinsic mean time to extinction*. Melbourne and Hastings (2008) showed that estimates of extinction risk depend strongly on *which* sources of stochasticity are included, because demographic heterogeneity and sex-ratio variation can dominate, so that risk attributed to the environment may in fact be demographic. Ovaskainen and Meerson (2010) reviewed the mathematics. Fagan and Holmes (2006) provided rare empirical evidence for vortex-like dynamics in declining vertebrate populations.

The parallel palaeontological tradition. Running alongside, and rarely cross-referencing the conservation literature, palaeontologists developed distinct senses of "stochastic." Raup and Sepkoski (1984) used it in a *time-series* sense, testing whether the 26-million-year periodicity of mass extinctions was a real signal or a chance pattern in the record, whereas Raup (1991) used it to mean extinction that is **non-selective with respect to traits**, the *field of bullets*: a claim about the *pattern* of who dies, not about whether the cause is random. Jablonski (2005; and Jablonski, 2008) examined when extinction is selective versus non-selective across mass-extinction boundaries.

These poles, the fully non-selective *field of bullets* versus trait-selective extinction, are not the whole story. Raup (1991) argued that the record is dominated by an intermediate mode, *wanton* extinction: **selective but "nonconstructive,"** in that the survivors are not the better-adapted ones. He traced it to **phylogenetic constraint**, the tendency of lineages toward anatomical stability, which limits how quickly they can evolve away from a novel stress. Jablonski (2005) later gave this a quantitative form: across mass-extinction boundaries most organism-level traits lose their power to predict survival, which instead tracks **geographic range at the clade level**, a "nonconstructive selectivity" decoupled from individual adaptedness. Both are reminders that a *pattern* of non-random survival need not imply selection on organismal fitness, and that the operative scale may be the clade rather than the individual.

Recent extensions. Work since 2010 has integrated genetics, demography, and landscape connectivity in single models (Benson et al., 2016); examined how mating systems and social structure modulate stochastic risk (Leach et al., 2020; Legendre et al., 1999); and linked stochasticity to evolutionary rescue and genetic erosion (Nordstrom et al., 2023). The *functional-extinction* literature (Jarić, 2015) approaches the same endpoint from the side of lost ecological function rather than lost individuals.

Table S1. The sequence in full. Every contribution discussed above, in the order it appeared. The final column separates the mathematical, ecological and conservation-biology line of descent from the parallel palaeontological one.

Year	Source	What it established	Strand
1939	Feller (1939)	Recast population dynamics as a birth-and-death process; extinction has positive probability at all sizes, and only because of the variance	Mathematics
1948	Kendall (1948)	Complete solution of the generalised birth-and-death process, with time-dependent rates, and the first explicit probability of ultimate extinction for that general case	Mathematics
1949	Bartlett (1949)	Unified the stochastic treatment across ecology and epidemiology	Mathematics
1957	Bartlett (1957)	Critical community size: the size below which measles cannot maintain its transmission cycle and stochastically fades out	Epidemiology
1967	MacArthur and Wilson (1967)	First major ecological application; mean time to extinction scales steeply with population size	Ecology
1969	Lewontin and Cohen (1969)	Under randomly varying growth it is the geometric, not the arithmetic, mean growth rate that governs long-term fate	Ecology
1972	Richter-Dyn and Goel (1972)	Bridged abstract birth-and-death theory and applied population biology	Mathematics and ecology
1981	Shaffer (1981)	Named and partitioned the sources (demographic, environmental, genetic, catastrophic) in the service of minimum viable populations	Conservation biology
1981	Leigh (1981)	Mean lifetime in a continuously varying environment, governed chiefly by the relative amplitude of fluctuation rather than by size	Ecology
1984	Raup and Sepkoski (1984)	"Stochastic" in the time-series sense: whether the periodicity of mass	Palaeontology

Year	Source	What it established	Strand
		extinctions is signal or a chance pattern in the record	
1986	Gilpin and Soulé (1986)	The extinction vortex: feedback among small size, stochasticity and genetic deterioration	Conservation biology
1988	Pimm and Redfearn (1988)	The measured variability of a population rises with the length of the census	Ecology
1991	Raup (1991)	The "field of bullets", extinction non-selective with respect to traits, and wanton extinction as the dominant intermediate mode	Palaeontology
1993	Lande (1993)	Canonical quantitative treatment of demographic and environmental stochasticity plus random catastrophes	Conservation biology
1999	Legendre et al. (1999)	Mating system and social structure modulate stochastic extinction risk	Ecology
2001	Inchausti and Halley (2001)	The same variability effect across 544 long series; spectra reddened, close to 1/f pink noise	Ecology
2004	Grimm and Wissel (2004)	The intrinsic mean time to extinction as a unifying currency for persistence measures	Ecology
2005	Jablonski (2005)	Across mass-extinction boundaries survival tracks geographic range at the clade level rather than organism-level traits	Palaeontology
2006	Fagan and Holmes (2006)	Rare empirical evidence for vortex-like dynamics in declining vertebrate populations	Conservation biology
2006	Pimm et al. (2006)	The Florida panther genetic rescue assessed after the fact; the effect was specific rather than general	Conservation biology
2008	Melbourne and Hastings (2008)	Estimated risk depends strongly on which sources are modelled; demographic heterogeneity and sex-ratio variation can dominate	Conservation biology
2010	Ovaskainen and Meerson (2010)	Review of the mathematics of stochastic population extinction	Mathematics
2015	Jarić (2015)	Functional extinction and cryptic function loss: the same endpoint approached from the side of lost ecological function	Conservation biology

Year	Source	What it established	Strand
2016	Benson et al. (2016)	Demography, genetics and landscape connectivity acting together in one wild large-carnivore population	Conservation biology
2019	Pimm et al. (2019)	Resilience as a measurement problem, with recovery time as the operational metric	Ecology
2020	Leach et al. (2020)	Social structure and mating system as sources of risk beyond the classic partition	Ecology
2021	Kong et al. (2021)	The two-class partition applied range-wide; genetic consequences are not modelled, so the reported risks are lower bounds	Conservation biology
2023	Nordstrom et al. (2023)	Density dependence, genetic erosion and the extinction vortex set against evolutionary rescue	Conservation biology

S2. The two classes, briefly

Class	What is random	Where it bites	Key references
**Environmental fluctuation** (external)	Vital rates, year to year, including rare severe years	All sizes; reduces geometric-mean growth	Lewontin and Cohen (1969); Lande (1993); Mangel and Tier (1994)
**Demographic accidents** (internal)	Which individuals die; how many are born, and of which sex; which alleles are transmitted	Small populations (the first two scale with $1/N$)	Shaffer (1981); Melbourne and Hastings (2008); Frankham (1995)

Two departures from the older four-way list of Shaffer (1981) and Lande (1993) deserve stating.

"Catastrophe" has no scale-free definition. Rare severe events are the extreme tail of the environmental distribution rather than a class of their own. Treating them separately requires a threshold for what counts as a catastrophe, and none is available: the measured variability of a population rises with the length of the census (Pimm and Redfearn, 1988), the increase decelerates in almost all series but generally fails to converge over ecologically observable timescales, and the spectra are reddened, with a mean redness exponent close to $1/f$ pink noise (Inchausti and Halley, 2001). Extremes therefore appear at every scale of observation, and "catastrophe" describes the observer's window as much as the event.

Genetic stochasticity is a demographic accident. Drift and inbreeding are what happens to allele frequencies when a finite number of individuals reproduce, and they belong with the vagaries of birth and death rather than in a category of their own. What separates the three subsets is not their origin but how well they can be modelled: the vagaries of death and of birth

follow from a life table, whereas the mapping from lost genetic variation to a change in survival, age at first reproduction or fecundity is not generally known in advance.

The three are not independent. The extinction vortex (Fagan and Holmes, 2006; Gilpin and Soulé, 1986) is precisely the statement that they feed back on one another: small size raises demographic and genetic stochasticity, which lowers growth and reduces size further.

S3. The scale (unit-of-reference) problem

The Explainer states that the stochastic/deterministic boundary is scale-dependent and that clarity requires naming the unit. That compression hides a genuinely hard issue, set out here.

The *same event* changes character with the level of description. A drought is a **deterministic driver** to a demographer modelling a single population's vital rates that year, but a **random shock** to a metapopulation model that does not resolve weather. Neither description is incorrect; they differ in what they hold fixed. Consequently:

- **Individual scale.** Randomness is the realised fate of each organism; "stochastic" = demographic stochasticity in its strict sense.
- **Population scale.** The classic partition applies most cleanly here, especially for vertebrates with long-term demographic data. The probability that a self-replacing population remains attainable is a statement at this scale.
- **Metapopulation scale.** Local extinctions are probabilistic events within a spatial network; persistence is a balance of local extinction and recolonisation (Hanski, 1998, 1999). A population that is "deterministically" doomed locally may persist regionally, and vice versa.
- **Clade / deep-time scale.** Here "stochastic" most often means *non-selective with respect to traits* (Raup, 1991), a pattern statement. Process-level stochasticity is rarely recoverable from the fossil record.

The practical consequence is that an unqualified claim such as "the extinction was stochastic" is under-determined. The viability-threshold reading advanced in the Explainer is explicitly a **population/metapopulation-level** statement; it is not intended as a claim about clades in deep time, where the Raup sense dominates.

S4. Pattern versus process, and the controversy

Two clarifications that the Explainer can only gesture at:

Pattern ≠ process. A stochastic *output* (e.g. extinctions distributed at random across taxa) does not require a stochastic *cause*; a deterministic cause (an impact) can produce a trait-non-selective pattern. Conversely, a deterministic *decline* (habitat loss) can have its *timing* set by demographic noise. The word "stochastic" can attach to either the cause or the pattern, and the two should not be conflated.

The viability definition is a deliberate position. Treating a lineage as effectively extinct once a viable, life-cycle-completing population is no longer attainable is more biologically

informative than waiting for the last individual, because it names *commitment to loss* (cf. extinction debt) rather than its final realisation. The cost is that it is harder to apply at the moment of classification and is sensitive to the chosen scale and horizon. We present it as the natural reading of long-standing population-biology practice, from Bartlett's critical community size to modern quasi-extinction thresholds, while acknowledging that a last-individual definition remains the more conservative and more auditable convention.

S5. Annotated key references

- **Feller (1939)**: origin of the birth-and-death process; conceptual ancestor of the whole field.
- **Bartlett (1957)**: critical community size and stochastic fade-out of measles; the earliest concrete viability-threshold idea.
- **MacArthur and Wilson (1967)**: first major ecological use; mean time to extinction scales with population size.
- **Lewontin and Cohen (1969)**: environmental stochasticity; geometric-mean growth governs fate.
- **Shaffer (1981)**: names the demographic/environmental/genetic partition; minimum viable populations.
- **Leigh (1981)**: approximate mean time to extinction in a varying environment; lifetime set by the relative amplitude of fluctuations, not just size.
- **Gilpin and Soulé (1986)**: the extinction vortex (feedback among sources).
- **Pimm and Redfearn (1988)**: the origin of "more time, more variation". The standard deviation of log density rises with the length of the census, for the majority of populations surveyed, which bears on whether populations have an equilibrium at all.
- **Raup (1991)**: the palaeontological "field of bullets"; non-selective pattern.
- **Lande (1993)**: canonical modern treatment of the partition + catastrophes.
- **Inchausti and Halley (2001)**: the same effect across 544 series (>30 yr, 123 species, median 46.5 yr) in the Global Population Dynamics Database. The increase decelerates in 96.9% of series but generally fails to converge over ecologically observable timescales; mean spectral redness exponent -1.022 (SE 0.025), close to 1/f pink noise.
- **Grimm and Wissel (2004)**: intrinsic mean time to extinction as a unifying currency.
- **Fagan and Holmes (2006)**: empirical evidence for vortex dynamics in vertebrates.
- **Pimm et al. (2006)**: the Florida panther genetic rescue, assessed after the fact. The effect was specific rather than general, which is why the consequences of lost genetic variability have to be established case by case.
- **Melbourne and Hastings (2008)**: extinction risk depends strongly on which sources are modelled; demographic factors can dominate.
- **Jarić (2015)**: functional extinction / cryptic function loss; the endpoint from the functional side.
- **Benson et al. (2016)**: first empirical demonstration of an extinction vortex in a wild large carnivore (Los Angeles pumas); demography, genetics, and landscape connectivity acting together.

- **Pimm et al. (2019)**: resilience is a measurement problem rather than a metaphor, and recovery time is the operational metric. The fractal structure of real time series is what makes "shock" and "recovery" hard to delimit.
- **Leach et al. (2020)**: social structure and mating system as a source of stochastic extinction risk; behaviour acting beyond the classic partition.
- **Kong et al. (2021)**: range-wide individual-based models for the giant panda, using the two-class partition of demographic accidents and environmental fluctuations. Genetic consequences are not modelled, so the reported risks are lower bounds.
- **Nordstrom et al. (2023)**: density dependence, genetic erosion, and the extinction vortex set against *evolutionary rescue*; the modern eco-evolutionary framing of the small-population problem.

References

Bartlett, M.S. (1949) Some evolutionary stochastic processes. *Journal of the Royal Statistical Society, Series B*, **11**, 211–229.

Bartlett, M.S. (1957) Measles periodicity and community size. *Journal of the Royal Statistical Society, Series A*, **120**, 48–70.

Bartlett, M.S. (1960) Stochastic population models in ecology and epidemiology. Methuen.

Benson, J.F., Mahoney, P.J., Sikich, J.A., Serieys, L.E.K., Pollinger, J.P., Ernest, H.B., and Riley, S.P.D. (2016) Interactions between demography, genetics, and landscape connectivity increase extinction probability for a small population of large carnivores in a major metropolitan area. *Proceedings of the Royal Society B*, **283**, 20160957.

Fagan, W.F. and Holmes, E.E. (2006) Quantifying the extinction vortex. *Ecology Letters*, **9**, 51–60.

Feller, W. (1939) Die Grundlagen der Volterraschen Theorie des Kampfes ums Dasein in wahrscheinlichkeitstheoretischer Behandlung. *Acta Biotheoretica*, **5**, 11–40.

Frankham, R. (1995) Conservation genetics. *Annual Review of Genetics*, **29**, 305–327.

Gilpin, M.E. and Soulé, M.E. (1986) Minimum viable populations: Processes of species extinction. in *Conservation biology: The science of scarcity and diversity*. Sinauer Associates.

Goel, N.S. and Richter-Dyn, N. (1974) Stochastic models in biology. Academic Press.

Grimm, V. and Wissel, C. (2004) The intrinsic mean time to extinction: A unifying approach to analysing persistence and viability of populations. *Oikos*, **105**, 501–511.

Hanski, I. (1998) Metapopulation dynamics. *Nature*, **396**, 41–49.

Hanski, I. (1999) Metapopulation ecology. Oxford University Press.

Inchausti, P. and Halley, J. (2001) Investigating long-term ecological variability using the Global Population Dynamics Database. *Science*, **293**, 655–657.

Jablonski, D. (2005) Mass extinctions and macroevolution. *Paleobiology*, **31**, 192–210.

Jablonski, D. (2008) Extinction and the spatial dynamics of biodiversity. *Proceedings of the National Academy of Sciences USA*, **105**, 11528–11535.

Jarić, I. (2015) Complexity and insidiousness of cryptic function loss mechanisms. *Trends in Ecology & Evolution*, **30**, 371–372.

Kendall, D.G. (1948) On the generalised 'birth-and-death' process. *Annals of Mathematical Statistics*, **19**, 1–15.

Kendall, D.G. (1949) Stochastic processes and population growth. *Journal of the Royal Statistical Society, Series B*, **11**, 230–282.

Kong, L., Xu, W., Xiao, Y., Pimm, S.L., Shi, H., and Ouyang, Z. (2021) Spatial models of giant pandas under current and future conditions reveal extinction risks. *Nature Ecology & Evolution*, **5**, 1309–1316.

Lande, R. (1993) Risks of population extinction from demographic and environmental stochasticity and random catastrophes. *The American Naturalist*, **142**, 911–927.

Leach, K., Kelly, R., Cameron, A., Montgomery, W.I., and Reid, N. (2020) Stochasticity in social structure and mating system drive extinction risk. *Ecosphere*, **11**, e03038.

Legendre, S., Clobert, J., Møller, A.P., and Sorci, G. (1999) Demographic stochasticity and social mating system in the process of extinction of small populations: The case of passerines introduced to New Zealand. *The American Naturalist*, **153**, 449–463.

Leigh, Jr., Egbert G. (1981) The average lifetime of a population in a varying environment. *Journal of Theoretical Biology*, **90**, 213–239.

Lewontin, R.C. and Cohen, D. (1969) On population growth in a randomly varying environment. *Proceedings of the National Academy of Sciences USA*, **62**, 1056–1060.

MacArthur, R.H. and Wilson, E.O. (1967) The theory of island biogeography. Princeton University Press.

Mangel, M. and Tier, C. (1994) Four facts every conservation biologist should know about persistence. *Ecology*, **75**, 607–614.

Melbourne, B.A. and Hastings, A. (2008) Extinction risk depends strongly on factors contributing to stochasticity. *Nature*, **454**, 100–103.

Nordstrom, S.W., Hufbauer, R.A., Olazcuaga, L., Durkee, L.F., and Melbourne, B.A. (2023) How density dependence, genetic erosion and the extinction vortex impact evolutionary rescue. *Proceedings of the Royal Society B*, **290**, 20231228.

Ovaskainen, O. and Meerson, B. (2010) Stochastic models of population extinction. *Trends in Ecology & Evolution*, **25**, 643–652.

Pimm, S.L., Dollar, L., and Bass, O.L. (2006) The genetic rescue of the Florida panther. *Animal Conservation*, **9**, 115–122.

Pimm, S.L., Donohue, I., Montoya, J.M., and Loreau, M. (2019) Measuring resilience is essential to understand it. *Nature Sustainability*, **2**, 895–897.

Pimm, S.L. and Redfearn, A. (1988) The variability of population densities. *Nature*, **334**, 613–614.

Raup, D.M. (1991) Extinction: Bad genes or bad luck? W. W. Norton.

Raup, D.M. and Sepkoski, J.J., Jr. (1984) Periodicity of extinctions in the geologic past. *Proceedings of the National Academy of Sciences*, **81**, 801–805.

Richter-Dyn, N. and Goel, N.S. (1972) On the extinction of a colonizing species. *Theoretical Population Biology*, **3**, 406–433.

Shaffer, M.L. (1981) Minimum population sizes for species conservation. *BioScience*, **31**, 131–134.
