# Supplementary material for: Explainer: Stochastic extinction
Source: Camb Prism Extinct. 2026 Oct 15;4:e16. doi: 10.1017/ext.2026.10023 (PMC13748990; doi:10.1017/ext.2026.10023)
Supplement: Peer review history

# Reviewer report: Review: Explainer: Stochastic extinction — R0/PR2

**DOI:** 10.1017/ext.2026.10023.pr2

**Authors:** Stuart Pimm

**Affiliations:** Duke University, United States

Explainer: stochastic extinction. (For Cambridge Prisms)

Writing an explainer of extinction processes for a likely audience of conservation practitioners is a very good idea. Bravo! That said, this effort has two sets of problems, one that will take modest effort to fix, the other will take rather more.

Conceptual problems.

The authors make a correct choice in recognising that human actions may cause inexorable declines to extinction in many species. We destroy their habitats, or we hunt them to extinction. Protecting species from these impacts has led to species recoveries.

2. The authors make a correct choice in recognising two kinds of stochastic effects. One is the nature of environments — broadly defined — external to the population under consideration. Extreme weather events are an obvious example. These can impact even large populations and often impact many species simultaneously.

3. The authors split these effects into two classes — separating out catastrophes from routine impacts. This is a mistake, and it begs the question of what constitutes a catastrophe.

One reason we’d like to know the difference is so that we can estimate a population’s “resilience” — meaning how fast a population can recover from a catastrophe. As the two examples in this paper show

Pimm S.L., Donohue I, Montoya J.M,. Loreau M. Measuring resilience is essential to understand it. Nature Sustainability. (2019) Oct;2(10):895-7.

this is far from simple. The authors should not separate two kinds of environmental stochasticity — especially putting them in separate classes as they do in lines 37 to 49.

Next, we come to demographic accidents. These are internal to the population, so peculiar to it, and are the unavoidable consequences of demography. As such, they form a class in themselves and one I would classify together, but with three subsets.

The vagaries of death: all or many of the individuals in a population dying in the same generation for independent reasons. If 20% of a population dies each generation, then a population of 1000 will be OK. But for a population of 10, and a Poisson lambda of survival of 0.8, there’s a chance that all the individuals will die each.

The vagaries of birth. Also unavoidable, with the chance that all those born in a generation will be of the same sex. This is a binomial process.

The loss of genetic variability and its consequences. The loss is well-understood — 1/Ne per generation where Ne is the effective population size. The consequences part is not well understood, in the sense that we don’t know how well a decline in genetic variability will translate into a reduction in adult life span or increased mortality of young, or decreased numbers of young or some combination of these.

Now, changing the authors’ groupings to the ones I suggested — which is a strong recommendation — is not going to take them much time. The problem is that once one recognises these, it becomes clear that the author’s Figure 1 is wrong. It suggests a threshold — a “cliff” over which a population falls. Rather, there is an area where there is a downhill slope that gets ever steeper, with the three demographic accidents creating a positive feedback spiral to extinction. (A “vortex”).

Practical problems.

I know these pieces have to be short, but I think the authors must be allowed a few words and — especially! — a few references to the practical consequences of all this.

All populations show the “more time means more variation” pattern. The longer we study a population, the more variable it becomes — not just measured by its extremes, but by its variance. (Pimm, S. L. & Redfearn, A. Nature 334, 613–614 (1988); Inchausti, P. & Halley, J. Science 293, 655–657 (2001).)

So deciding between a population that is showing a long-term decline from human impacts and one that is showing a mid-term decline, likely to be reversed, as part of its natural changes is not an easy one. The implications for conservation are profound. Generally, one needs external information about those human impacts, to identify them and stop them.

In the example of the Illinois prairie chicken, those who managed it tried many interventions before they finally accepted the population was inbred and they needed a genetic rescue.

Second, because of the fractal nature of the “more time means more variation” pattern, it can be hard to identify “catastrophes” as a separate class of environmental variation.

Deciding between the vagaries of birth and death and the loss of genetic variation and its consequences is difficult. (As explained, one can model the first two, but not the second.) So I had argued that a genetic rescue of the Florida panther was pointless: those vagaries plus the increasing loss of habitat would doom it. In the fullness of time, the National Park Service asked me to review the genetic rescue, and I retracted that opinion. Pimm SL, Dollar L, Bass Jr OL. The genetic rescue of the Florida panther. Animal Conservation. 2006 May;9(2):115-22. (The genetic effects had a major impact on the survival of the kittens, but not the adult demographics.)

Finally, because one cannot easily model the consequences of small populations on lost fecundity, one may model just the demographic issues. One can notice that these paint too rosy a picture, and demographc interventions may still be essential. Kong, L., Xu, W., Xiao, Y., Pimm, S.L., Shi, H. and Ouyang, Z., 2021. Spatial models of giant pandas under current and future conditions reveal extinction risks. Nature Ecology & Evolution, pp.1-8.

The authors may well have other case histories to discuss — and that’s fine. But, they must connect these theoretical musing with the practice, but to clarify their theories and to make the piece useful

# Reviewer report: Review: Explainer: Stochastic extinction — R0/PR3

**DOI:** 10.1017/ext.2026.10023.pr3

An excellent job of covering the definition of stochastic extinction.

- Line 39 – consider changing ‘shuffling of genes’ to ‘shuffling and loss of genes’

- All Figures – In this new world of stating when generative AI is used, I see no need to acknowledge its help with coding. We did not acknowledge Google or Stackoverflow for the same use.

- Fig 2 – text needs to be larger. Same for much of Fig 3.

- Line 40 – Is it when ‘self-replacing population is not longer attainable’, or when the probability of persistence for some amount of time is sufficiently low? This seems like a tricky thing to state clearly because it is a probability problem.

- Line 44 – Failing to find a mate is one mechanism that can cause an Allee effect, so I’m not sure why they are listed separately.

# Editor report: Recommendation: Explainer: Stochastic extinction — R0/PR4

**DOI:** 10.1017/ext.2026.10023.pr4

**Affiliations:** Durrell Institute of Conservation and Ecology, University of Kent, United Kingdom of Great Britain and Northern Ireland

Both reviewers have provided useful comments regarding the manuscript. Review 1 has provided some suggestions regard theoretical and practical issues around stochastic extinction that you may want to considered to help improve the precision of its use and illustrate its application. That said the idea of the ‘Explainer’ is to provide something short paper for those new to the field and/or outside of the field such as journalists and politicians. I would therefore prefer if the manuscript could be kept within the word limit as best you can and where necessary, and as you already have, make use of supplementary materials as my concern anything too long will reduce the audience for this important term.

# Editor report: Decision: Explainer: Stochastic extinction — R0/PR5

**DOI:** 10.1017/ext.2026.10023.pr5

**Affiliations:** School of Natural Sciences, University of Tasmania, Australia

No accompanying comment.

# Editor report: Recommendation: Explainer: Stochastic extinction — R1/PR7

**DOI:** 10.1017/ext.2026.10023.pr7

**Affiliations:** Durrell Institute of Conservation and Ecology, University of Kent, United Kingdom of Great Britain and Northern Ireland

Having read the careful response to the reviews, manuscript and supplementary materials I am happy to recommend the manuscript be accepted. It is clear that you have taken the comments and board and strengthened the manuscript that I now look forward to seeing it published.

# Editor report: Decision: Explainer: Stochastic extinction — R1/PR8

**DOI:** 10.1017/ext.2026.10023.pr8

**Affiliations:** School of Natural Sciences, University of Tasmania, Australia

No accompanying comment.
